# Collision indicator charts for gantry‐couch position combinations for Siemens ONCOR and Elekta Infinity linacs

**DOI:** 10.1120/jacmp.v14i5.4355

**Published:** 2013-09-06

**Authors:** Stewart J. Becker, Wes Culberson, Ryan T. Flynn

**Affiliations:** ^1^ Radiation Oncology NYU Langone Medical Center New York NY USA; ^2^ Santa Cruz Radiation Oncology Santa Cruz CA USA; ^3^ Radiation Oncology University of Iowa Hospitals and Clinics Iowa City IA USA

**Keywords:** collision, treatment planning, gantry, couch, noncoplanar, Siemens ONCOR, Elekta Infinity

## Abstract

Noncoplanar radiation fields from a linear accelerator can be used to deliver radiation dose distributions that are superior to those delivered using coplanar radiation fields. Noncoplanar radiation field arrangements are especially valuable when delivering stereotactic body radiation therapy (SBRT). Noncoplanar radiation fields, however, are geometrically more challenging to deliver than coplanar radiation fields, and are associated with a greater risk of collisions between the gantry, treatment couch, and patient. Knowledge of which treatment couch offset, treatment couch angle, and gantry angle combinations provide a collision‐free radiotherapy delivery is useful in the treatment planning process, as the risk of requiring replanning due to improperly selected treatment parameters can be minimized. Such tables are by default specific to the linear accelerator make and model used for treatment. In this work a set of plots is presented indicating which combination of treatment couch lateral offsets (‐10 cm to 10 cm), couch angles (270° to 90°), and gantry angles (0° to 360°), will result in collision‐free radiation delivery using Siemens ONCOR linear accelerators equipped with a 160‐leaf multileaf collimator and a 550 TxT treatment table, and a Elekta Infinity linear accelerator with an MLCi2 and Elekta iBEAM evo Couchtop EP. The patient was assumed to have a width of 50 cm and a height of 25 cm.

PACS numbers: 87.55.‐x, 87.56.‐v

## I. INTRODUCTION

Noncoplanar radiation fields are useful for minimizing the volume of normal tissue receiving high doses in patients receiving stereotactic body radiotherapy (SBRT).^(1)^ In the external beam radiotherapy treatment planning process, it can be challenging to predict when collisions will occur between the linear accelerator gantry, treatment couch, and patient, especially when noncoplanar radiation fields are used. As reported in a previous paper on Varian linear accelerators (Palo Alto, CA),^(2)^ small variations in treatment couch heights and lateral offsets can dramatically affect which gantry and treatment couch angles can be utilized for a given radiation field. In the current work, plots of gantry angle, treatment couch lateral offset, height, and angle combinations that are collision‐free are presented for Siemens ONCOR (Erlangen, Germany) and Elekta Infinity (Stockholm, Sweden) linear accelerators.

## II. MATERIALS AND METHODS

The measurements presented were acquired on two different accelerator platforms. The first was a Siemens ONCOR linear accelerator with a 160‐leaf multileaf collimator and a 550 TxT treatment couch using methods described previously.^(2)^ The second was an Elekta Infinity with an MLCi2 and iBEAM evo Couchtop EP. All gantry and treatment couch angles are expressed relative to an observer looking at the gantry from the foot of the treatment couch when it is in the 0° position. The treatment couch is at 0° when it is aligned parallel to the axis of gantry rotation, 90° when rotated to the observer's right, and 270° to the observer's left. The treatment couch can be offset to the observer's right (positive) and left (negative). The gantry angle is 0° when the radiation field is pointing vertically down, and the gantry angle increases as the gantry rotates clockwise relative to its axis. This system corresponds to the IEC 1217, with the exception of the couch vertical which is expressed as positive going down compared to negative (in IEC 1217). Treatment couch positions were measured between 90° and 270° crossing through 0°. Treatment couch vertical positions of 10, 15, and 20 cm below the axis of gantry rotation and lateral treatment couch offsets of 0 and ± 10 cm were selected since they encompass the majority of clinical situations. Symmetry was verified for both machines (e.g., gantry 75°, couch 30° is equal to gantry 285°, couch 330°).

The maximum couch angle achievable for each gantry position was determined by first rotating the gantry to a position and then rotating the couch such that it was within 1° of colliding with the gantry. Measurements were taken at higher sampling rate near collision zones, and the gantry angle sampling frequency was at least 5°. For the Siemens platform, the patient's presence was simulated by placing typical vacuum‐based immobilization systems on the treatment couch, which had a height of 25 cm and a width of 50 cm. For the Elekta platform, an Eelkta BodyFix immobilization system was used with the compression paddle attachment in place. Any near‐collisions with the immobilization systems were assumed to be patient collisions also.

## III. RESULTS

**Figure 1:**
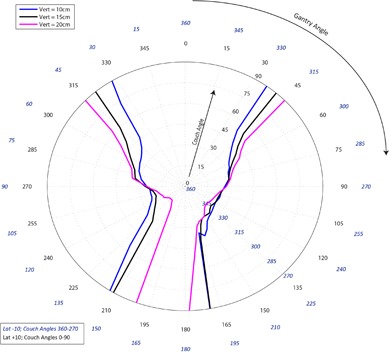
Siemens couch vs. gantry chart for vertical couch positions of 10 cm (blue), 15 cm (black), and 20 cm (magenta), lateral offset of 0 cm, and couch rotations from 0° to 90° (black) and 360°‐270° (blue). Every angle inside the lines (including the origin) is collision free.

**Figure 2:**
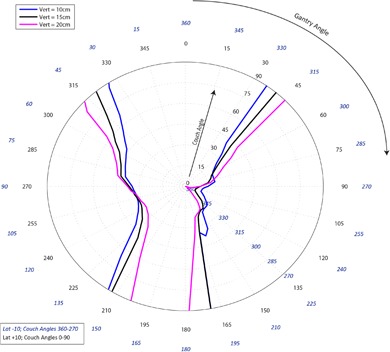
Siemens couch vs. gantry chart for vertical couch positions of 10 cm (blue), 15 cm (black), and 20 cm (magenta), lateral offsets of 10 cm in the direction of the couch angle swing. Every angle inside the lines (including the origin) is collision free.

**Figure 3:**
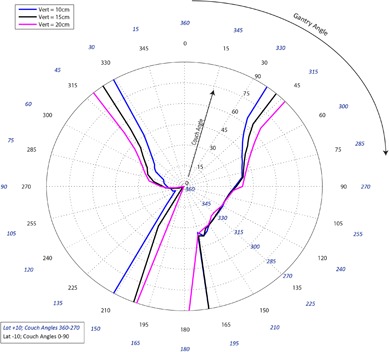
Siemens couch vs. gantry chart for vertical couch positions of 10 cm (blue), 15 cm (black), and 20 cm (magenta), lateral offsets of 10 cm in the opposite direction of the couch angle swing. Every angle inside the lines (including the origin) is collision free.

**Figure 4:**
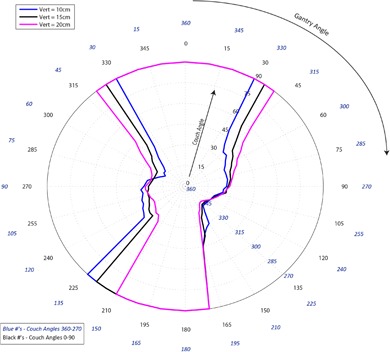
Elekta couch vs. gantry chart for vertical couch positions of 10 cm (blue), 15 cm (black), and 20 cm (magenta), lateral offset of 0 cm, and couch rotations from 0° to 90° (black) and 360°‐270° (blue). Every angle inside the lines (including the origin) is collision free.

**Figure 5:**
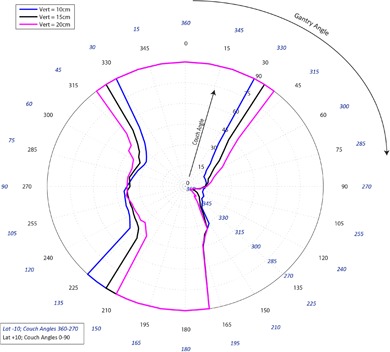
Elekta couch vs. gantry chart for vertical couch positions of 10 cm (blue), 15 cm (black), and 20 cm (magenta), lateral offsets of 10 cm in the direction of the couch angle swing. Every angle inside the lines (including the origin) is collision free.

**Figure 6:**
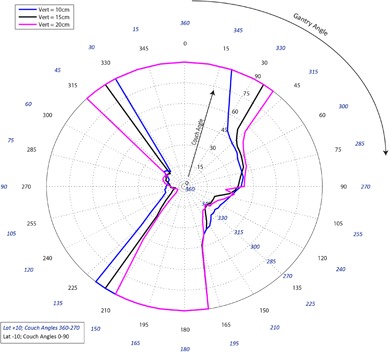
Elekta couch vs. gantry chart for vertical couch positions of 10 cm (blue), 15 cm (black), and 20 cm (magenta), lateral offsets of 10 cm in the opposite direction of the couch angle swing. Every angle inside the lines (including the origin) is collision free.

Figures 1–3 pertain to treatment couch lateral offsets of 0 cm, +10 cm, and ‐10 cm, respectively, for a Siemens linac and couch, and show the corresponding allowable gantry angles, treatment couch angles, and treatment couch heights. Figures 4–6 show similar data for an Elekta linac/couch combination. The area inside the curves, including the origin, is the “collision free” zone.

## IV. DISCUSSION & CONCLUSIONS

The most conservative estimate for a collision‐free couch angle at a given gantry angle, treatment couch height and treatment couch offset may be read from the appropriate plot by finding the gantry angle on the outside of the polar plot and tracing toward the origin to find the treatment couch angle. The most conservative estimate for safe gantry‐couch angle combination is not always associated with the same couch height, which reflects consideration for anterior versus posterior oblique gantry angles.

When the couch is rotated towards the gantry, the gantry always collides with the base of the couch regardless of which vertical treatment couch position is used. For treatment couch heights of 20 cm below isocenter, the collision occurs sooner than for 15 cm or less due to the presence of pendant holders on the left and right of the Siemens 550 TXT couch. For instance in Fig. 1, the couch and gantry do not collide at couch height of 10 cm or 15 cm when the gantry is 175° and a couch angle is 90°; however, if the couch height is dropped to 20 cm, there will be a collision at that same gantry and couch angles.

The Elekta couch has the option to fold the pendant out of the way and, therefore, it behaves in a much more predictable manner.

## ACKNOWLEDGMENTS

The authors wish to thank Celeste Leary from Santa Cruz Radiation Oncology for helping perform the measurements.
